# A chromosome-level genome assembly of yellow stem borer (*Scirpophaga incertulas*)

**DOI:** 10.1038/s41597-024-03108-3

**Published:** 2024-03-08

**Authors:** Sicong Zhou, Guanghua Luo, Qiong Yang, Yangchun Han, Kaili Yuan, Rui Ji, Jichao Fang

**Affiliations:** 1 Institute of Plant Protection, Jiangsu Academy of Agricultural Sciences, Jiangsu Key Laboratory for Food and Safety-State Key Laboratory Cultivation Base of Ministry of Science and Technology, Nanjing, 210014 China; 2 Bio21 Institute, School of BioSciences, University of Melbourne, Parkville, Victoria 3010 Australia; 3 Integrated Technical Service Center of Jiangyin Customs, Jiangyin, 214441 China; 4 Jiangsu Collaborative Innovation Center of Regional Modern Agriculture & Environmental Protection, Huaiyin Normal University, Huaian, 223300 China

**Keywords:** Genome assembly algorithms, Entomology

## Abstract

The yellow stem borer *Scirpophaga incertulas* is the dominant pest of rice in tropical Asia. However, the lack of genomic resources makes it difficult to understand their invasiveness and ecological adaptation. A high-quality chromosome-level genome of *S. incertulas*, a monophagous rice pest, was assembled by combining Illumina short reads, PacBio HiFi long sequencing, and Hi-C scaffolding technology. The final genome size was 695.65 Mb, with a scaffold N50 of 28.02 Mb, and 93.50% of the assembled sequences were anchored to 22 chromosomes. BUSCO analysis demonstrated that this genome assembly had a high level of completeness, with 97.65% gene coverage. A total of 14,850 protein-coding genes and 366.98 Mb of transposable elements were identified. In addition, comparative genomic analyses indicated that chemosensory processes and detoxification capacity may play critical roles in the specialized host preference of *S. incertulas*. In summary, the chromosome-level genome assembly of *S. incertulas* provides a valuable genetic resource for understanding the biological characteristics of its invasiveness and developing an efficient management strategy.

## Background & Summary

**Fig. 1:**
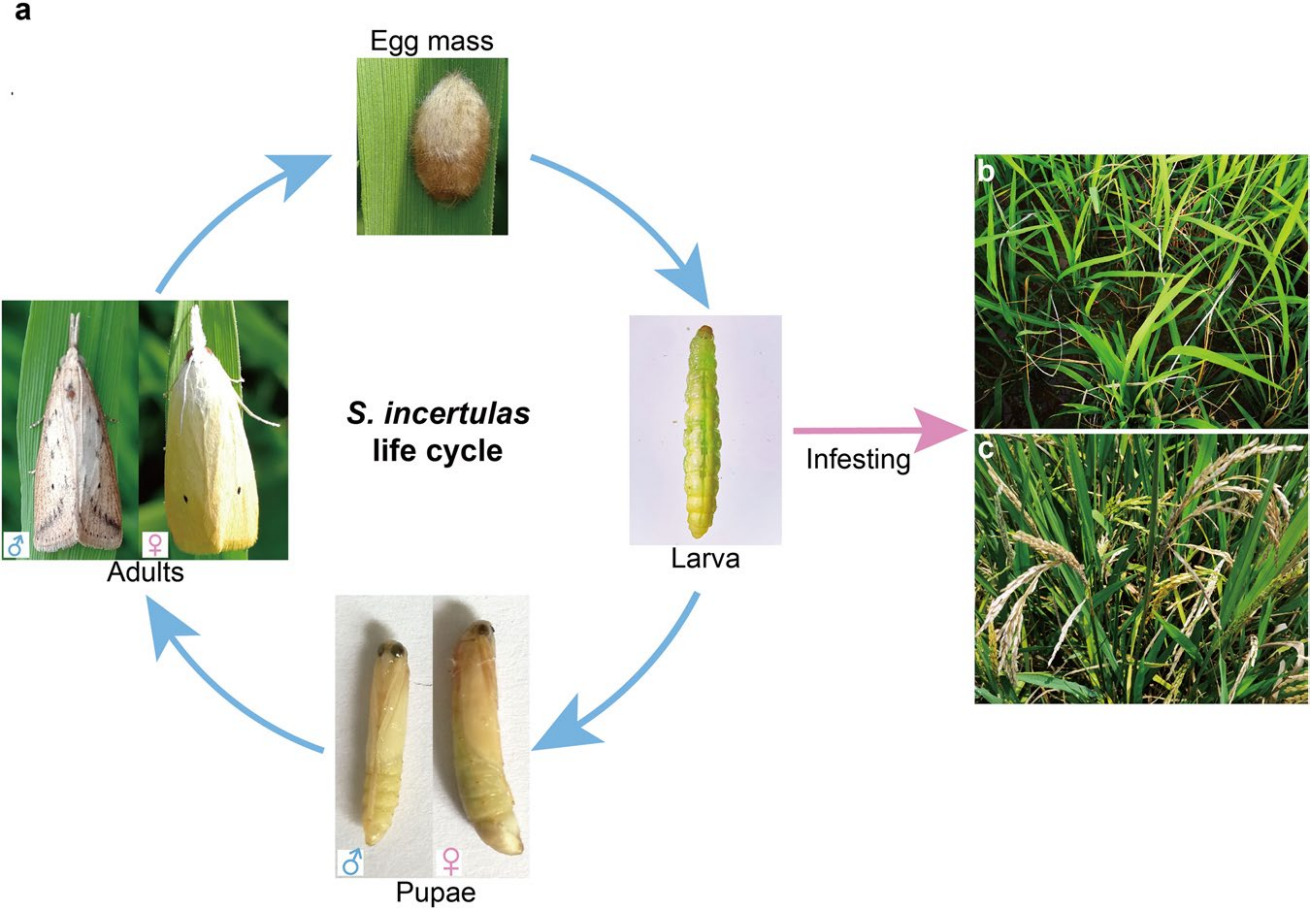
Development cycle and damage of *S. incertulas*. (**a**) Different developmental stages of *S. incertulas*. (**b**) Dead heart in the rice vegetative stage damaged by *S. incertulas*. (**c**) White ears in the rice reproductive stage damaged by *S. incertulas*.

Rice (*Oryza sativa* L.) is one of the world’s most important crops, providing a staple food for nearly half of the global population^1^. Insect pests continue to be a major threat to rice production, and stem borers are key pest species. Yellow stem borer (YSB), *Scirpophaga incertulas* (Lepidoptera: Crambidae), is the most destructive one found in diverse ecosystems across the world^2^. YSB has been reported to be the dominant pest in Asia^3^, Southeast Asia^4^, China, and India in particular^5^. Unlike another stem borer, *Chilo suppressalis* (which can feed on rice, maize, broomcorn millet, and wheat), *S. incertulas* is predominantly a monophagous pest, and there are no reports that it can successfully complete its life cycle on any plant outside *Oryza* species^6^. *S. incertulas* attacks rice plants from the seedling to maturity stages, and newly emerged larvae enter the stem to feed on the internal tissues at the vegetative and reproductive stages of plant growth, resulting in the formation of dead hearts and white ears (Fig. 1b,c). The yield loss caused by YSB may vary from 10% to 90%^6–8^ depending on the stage of the rice at which the insect attacks. With continuous high-quality and high-yield rice production in China, pest resurgence has become too serious to be ignored in recent years. The application of chemical insecticides during the seedling and reproductive stages of rice is a widely adopted practice for the management of *S. incertulas*. However, after repeated application of insecticides, it is difficult to manage YSB because of their increased resistance^9^. Moreover, the continuous use of pesticides poses health and environmental hazards^10^. Therefore, it is crucial to develop alternative strategies for managing this pest. Understanding the biochemical and molecular mechanisms of YSB’s invasiveness is critical for its control; however, such studies are hampered by a lack of high-quality genomic resources.

**Fig. 2:**
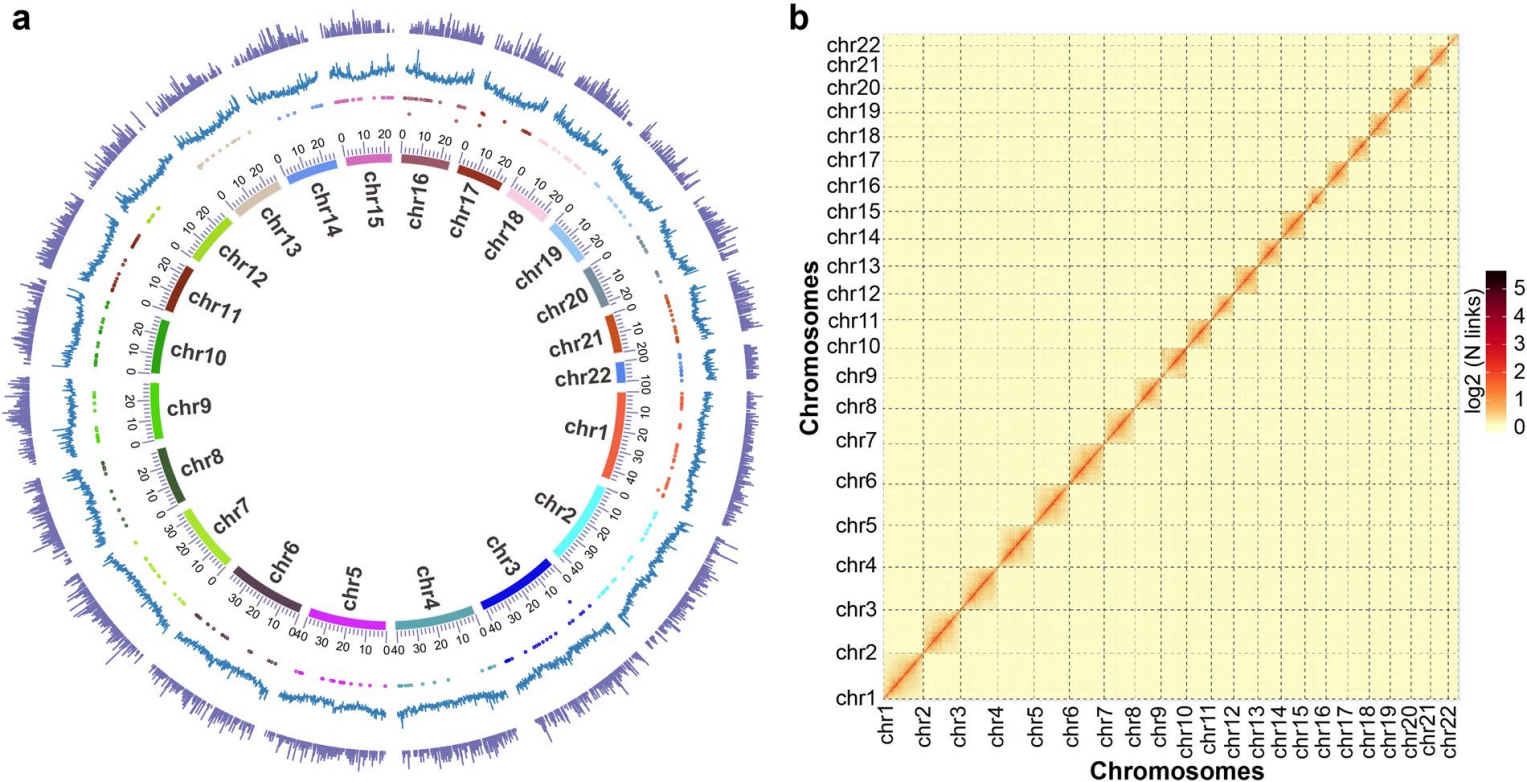
Genome assembly of *S. incertulas*. (**a**) Circle genome landscape of *S. incertulas*. Blocks on the innermost circle represent all 22 chromosomes of *S. incertulas*. Peak plots from inner to outer circles represent: N (unknown base) ratio, GC content, and gene density, respectively. (**b**) Hi-C interactive heatmap of *S. incertulas*. The color indicates the intensity of the interaction signal, with a darker color indicating a higher intensity.

In this study, we used short reads generated by an Illumina platform, long reads generated by PacBio sequencing, and high-throughput chromosomal conformation capture (Hi-C) analysis to construct a high-quality *S. incertulas* reference genome at the chromosomal level (Table 1). The genome sequences were assembled into 1,650 contigs, with a contig N50 length of 3.15 Mb and a total length of 695.65 Mb (Table 2). Chromosome scaffolding resulted in 1,299 sequences corresponding to 22 chromosomes, with a scaffold N50 of 28.02 Mb (Fig. 2a; Table 3). These results indicate a significantly improved genome assembly of *S. incertulas* than a recent report based on short-read sequencing^11^ (Table 2). We also identified 366.98 Mb of repeating sequences accounting for 52.75% of the genome assembly (Table 4). A total of 14,850 protein-coding genes were identified, of which 95.27% were annotated (Table 5).

**Table 1:** Sequencing data generated for the *S. incertulas* genome assembly and annotation.

Platform	Molecule	Clean data (Gb)	Usage	SRA accession number
Illumina HiSeq	DNA	67.41	correction	SRR27108979
PacBio HiFi	DNA	65.65	genome assembly	SRR27108978
Illumina Hi-C	DNA	37.98	chromosome-level assembly	SRR27204786
Illumina HiSeq	RNA	11.40	gene structure annotation	SRR27108980

**Table 2:** Comparative statistics of two *S. incertulas* genome assemblies.

Species	*S. incertulas*	*S. incertulas*^11^
	(This study)	(Kattupalli *et al*.)
Genome size (MB)	695.65	308.44
Number chromosomes	22	—
Contig number	1,650	310,612
Contig N50 (Kb)	3,150	1.26
Scaffold number	1,299	—
Scaffold N50 (Mb)	28.02	—
BUSCO complete rate of the genome (%)	97.65	48.87
GC content (%)	35.57	36.37
Protein-coding genes	14,850	46,057
Repeat (%)	52.80	—

**Table 3:** Statistics for *S. incertulas* genome sequence length (chromosome level).

Chromosome	Chromosome size (bp)
1	44,858,671
2	42,418,323
3	41,714,049
4	41,019,006
5	40,091,318
6	39,407,372
7	34,610,075
8	29,851,628
9	29,089,143
10	28,019,431
11	25,263,117
12	27,268,047
13	26,591,446
14	26,809,232
15	23,840,302
16	25,005,516
17	24,178,740
18	23,505,882
19	23,663,185
20	22,005,738
21	20,160,890
22	11,100,499

**Table 4:** Repetitive sequences in the *S. incertulas* genome assembly.

Type	Number	Length (bp)	Percentage (%)
LTR	76l,081	54,454,332	7.83
LINE	583,953	150,275,433	21.60
SINE	1,419	341,769	0.05
LARD	273,987	61,517,701	8.84
DNA transposons	289,664	79,734,828	11.46
Others	58,362	20,658,139	2.97
Total	1,283,466	366,982,202	52.75

**Table 5:** Functional annotation of the *S. incertulas* genome assembly.

Annotation database		Number	Percentage in genome (%)
Protein-coding genes		14,850	100
All Annotated		14,148	95.27
	GO	7,446	50.14
	KEGG	5,762	38.80
	KOG	9,268	62.41
	Pfam	11,067	74.53
	Swissprot	8,480	57.10
	TrEMBL	14,085	94.85
	Nr	14,061	94.69

**Fig. 3:**
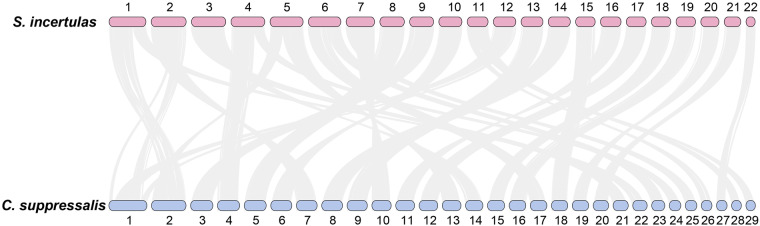
Chromosome-level synteny analysis. Chromosome-level synteny analysis of *S. incertulas* and another rice stem borer, *C. suppressalis*.

**Fig. 4:**
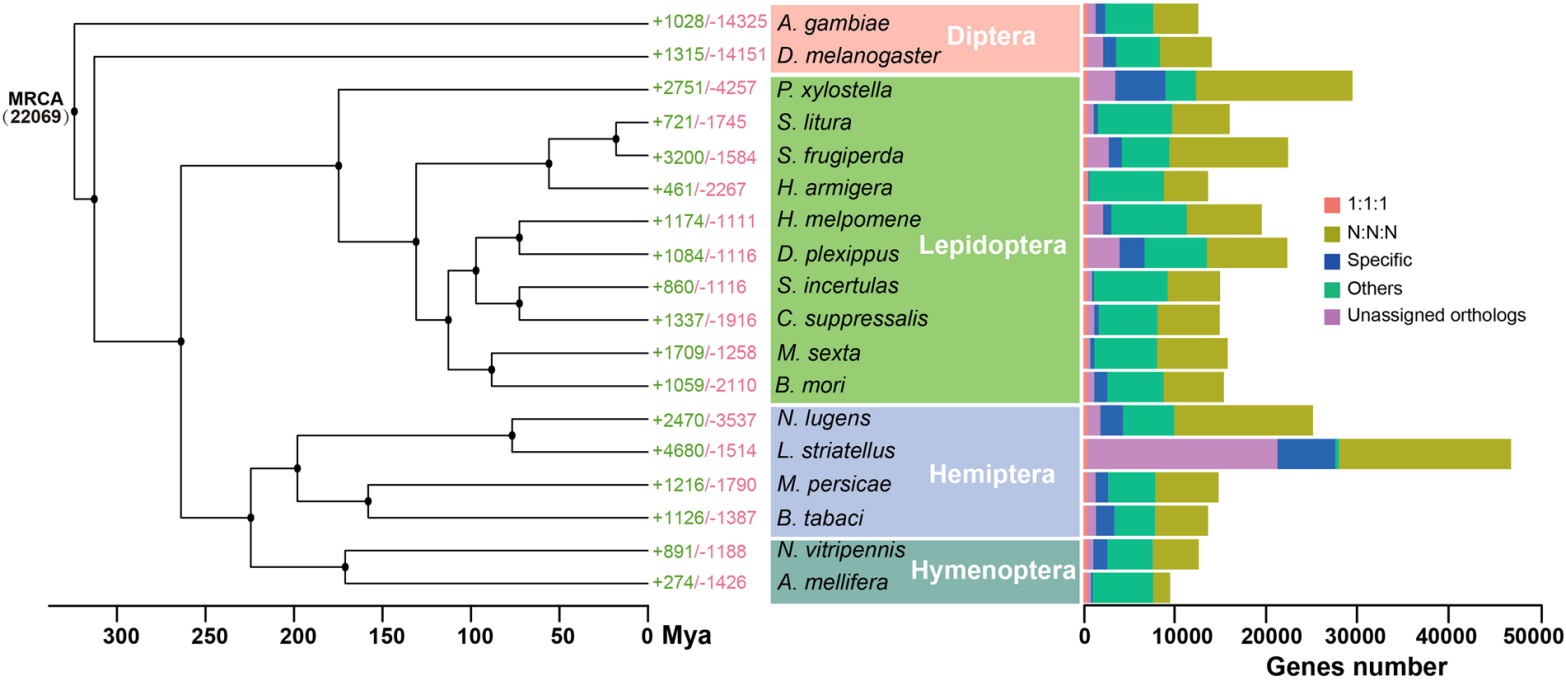
Phylogenetic tree of *S. incertulas* together with 17 other insects. A phylogenetic tree of *S. incertulas* and other 17 insect species was constructed using the maximum likelihood method with concatenated protein sequences of 415 single-copy orthologous genes with 1,000 bootstrap replicates. The numbers of expanded gene families (green) and contracted gene families (red) are shown to the right of each species branch. MRCA is the most recent common ancestor. The colored histogram indicates that the genes of each species were categorized into five groups: 1:1:1 (single-copy orthologous genes in common gene families); N:N:N (multiple copy orthologous genes in common gene common gene families); Specific (genes from unique gene families from each species); Others (genes that do not belong to any of the above ortholog categories); Unassigned orthologs (genes that were not clustered into any family).

The YSB genome showed high chromosomal synteny with *C. suppressalis* (Fig. 3), and phylogenetic analysis revealed that *S. incertulas* diverged from *C. suppressalis* approximately 72.65 million years ago (Mya) (Fig. 4). Furthermore, 860 expanded and 1,116 contracted gene families were identified in the *S. incertulas* genome compared to the common ancestor of *S. incertulas* and *C. suppressalis* (Fig. 4). Gene Ontology (GO) enrichment analysis of the expanded gene families showed that these genes were significantly enriched in the “defense responses to bacterium”, “biotic stimulus”, and “other organism” terms, which might play critical roles in increasing the ecological adaptation and insecticide resistance of YSB (Supplementary Table 3).

**Fig. 5:**
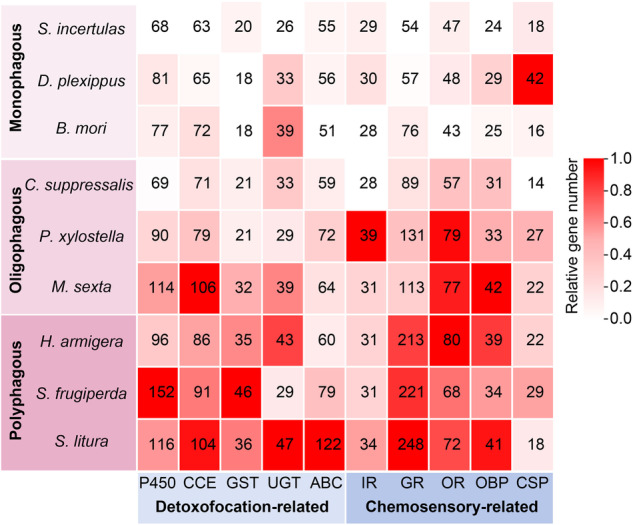
Distribution of detoxification and chemosensory genes in *S. incertulas* and eight other Lepidopteran insects. The numbers in the cells indicate the scale of the corresponding gene family for each species. A darker background color of the cells indicates that more genes were encoded in the corresponding species.

Based on the host-plant selection range, the feeding preferences of phytophagous insects are classified as monophagous, oligophagous, or polyphagous. The different host ranges may be reflected in the genome, and it has been previously suggested that detoxification capabilities and chemosensory processes are critical for host-plant selection in phytophagous insects^12–16^. In our study, we observed a positive relationship between the number of protein-coding genes and the host range (Fig. 5). Among the detoxification-related genes, the gene family sizes of cytochrome P450 (P450), carboxyl/choline esterase (CCE), and glutathione-S-transferase (GST) increased sequentially in monophagous, oligophagous, and polyphagous insects (Fig. 5). In addition, relatively high numbers of chemosensory-related genes for gustatory receptors (GRs), odorant receptors (ORs), and odorant-binding proteins (OBPs) were identified in insects with broader host ranges (Fig. 5). The numbers of protein-coding genes belong to GRs, ORs, and OBPs identified in *S. incertulas* were 54, 47, and 24, respectively, which were less than the number in *C. suppressalis* and largely less than the number in polyphagous species. Our results indicate a correlation between the relatively low numbers of detoxification and chemosensory-related genes identified in YSB and its specialized feeding preference (Fig. 5). The high-quality chromosome-level genome assembly of *S. incertulas* provides a valuable genomic resource for understanding the genetic, evolutionary, and ecological issues of YSB, and further offers the possibility to implement integrated pest management of this monophagous pest.

## Methods

### Sample collection and genome sequencing

The fifth instar larvae of *S. incertulas* were collected from rice (*Oryza sativa* L.) fields in Guangnan County, Yunnan Province, China. To decrease the level of sequencing heterozygosity, the number of insects used for sequencing was minimized. Genomic DNA was extracted from a single surface-sterilized fifth instar larva using the QIAamp DNA Mini Kit (Qiagen, Hilden, German) for Illumina, PacBio, and Hi-C sequencing, respectively. The purity and integrity of the genomic DNA were validated using a NanoDrop 2000C spectrophotometer (Thermo Fisher Scientific, Wilmington, DE, USA) and 1.5% agarose gel electrophoresis. Two independent paired-end libraries with a 270 bp inserted fragment were constructed and sequenced on an Illumina HiSeq 4000 platform following the manufacturer’s instructions (Biomarker Technologies Co., Ltd, Beijing, China). After removing adapter sequences and low-quality reads using HTQC (v1.92.3) software^17^, 67.41 Gb of clean data were obtained for subsequent analyses (Table 1). For PacBio HiFi sequencing, genomic DNA was sheared into ~15 kb fragments using g-Tubes (Covaris, Woburn, MA, USA) and purified using 0.45 × AMPure PB beads (Beckman Coulter, Brea, CA, USA) to construct SMRT bell libraries. Size selection was performed using BluePippin (Sage Science, Beverly, MA, USA) to collect 15–18 kb fragments. After annealing the primers and binding Sequel DNA polymerase to SMRT bell templates, sequencing was performed using one SMRT cell 1 M on the Sequel System (Biomarker Technologies). Finally, a total of 65.65 Gb of subreads were obtained, with an average read length of 10.43 kb, resulting in 94.37 × coverage of the *S*. *incertulas* genome (Table 1). To achieve chromosome-level assembly, the Hi-C technique was used to identify contacts between different regions of chromatin filaments. The Hi-C library was constructed following the standard library preparation protocol^18^ and sequenced on the Illumina HiSeq 4000 platform, and 37.98 Gb of 150-bp paired-end clean reads were obtained.

### RNA extraction and transcriptome sequencing

Five fifth instar *S*. *incertulas* larvae collected from rice (*Oryza sativa* L.) fields in Guangnan County were used for RNA extraction. Total RNA was extracted using TRIzol reagent (Invitrogen, Carlsbad, CA, USA) following the manufacturer’s instructions. The concentration of the isolated RNA was measured using a NanoDrop 2000C spectrophotometer (Thermo Fisher Scientific). RNA quality was evaluated using 1.5% agarose gel electrophoresis. RNA integrity was quantified using an Agilent 5400 Fragment Analyzer (Agilent, Santa Clara, CA, USA). RNA-seq libraries were constructed using the NEBNext® Ultra™ RNA Library Prep Kit (NEB, Ipswich, MA, USA) following the manufacturer’s instructions. Libraries were then sequenced on the Illumina Hiseq 4000 platform (Biomarker Technologies), and 11.40 Gb of 150-bp paired-end reads were obtained and used for gene prediction (Table 1).

### Genome estimation and assembly

A genome survey is essential for estimating the main genome characteristics, including genome size, repetitive sequence content, and heterozygosity. The k-mer (K = 19) frequencies were constructed based on Illumina clean short reads using Jellyfish (v2.2.10)^19^ and were used to perform a genome survey using GenomeScope (v2.0)^20^. Heterozygosity revealed by k-mer analysis reflects the inner heterozygosity of an individual. As a result, the estimated genome scale of *S*. *incertulas* was 673.86 Mb, with a heterozygosity rate of 1.03%, a repeat ratio of 45.97%, and a GC content of 37.36% (Supplementary Figure 1). PacBio long-read data were used to generate a contig-level assembly of the *S. incertulas* genome. A preliminary assembly was generated using WTDBG2 (v2.5)^21^ with the default parameters. After correcting for short-read using Pilon (v1.23)^22^, the *S. incertulas* genome assembly was generated, which consisted of 1,650 contigs with a total length of 695.65 Mb and a contig N50 of 3.15 Mb (Table 2). After removing the low-quality reads and adaptor sequences, 37.98 Gb of clean data were generated from the Hi-C library and mapped to the draft *S. incertulas* genome using BWA (v0.7.10)^23^ with the default parameters. Uniquely aligned read pairs were further processed using HiC-Pro (v2.10.0)^24^ to assess and eliminate invalid read pairs, including dangling ends, re-ligation, self-cycle, and dumped pairs. A total of 21,595,092 valid interaction pairs for scaffold correction were used to cluster, order, and orient the contigs onto chromosomes using LACHESIS (v2e27abb)^25^ with the default parameters. Ultimately, 373 sequences were anchored to 22 chromosomes with a scaffold N50 of 28.02 Mb, covering a span of 650.46 Mb and representing 93.50% of the draft genome assembly (Fig. 2a; Table 2). The lengths of the 22 chromosomes ranged from 11.10 Mb to 44.86 Mb (Fig. 2a; Table 3). The scaffold N50 in our assembly was much higher than the 1.26 Kb in another recent version of *S. incertulas* (Table 2).

### Genomic repeat annotation

Repeat sequences mainly include tandem and interspersed repeats, the latter being primarily transposable elements (TEs). The repeat TE sequences were annotated using a combination of homology-based and *de novo* approaches. We initially customized a *de novo* repeat library using RepeatModeler (v2.0.2a)^26^ and LTR_retriever (v2.8)^27^ based on assembly sequences with default parameters. The predicted repeats were subsequently classified using the PASTE Classifier (v1.0)^28^, and the results were combined with the database of Dfam (v3.2)^29^ to construct a species-specific TE library without redundancy. The TE sequences were identified by homology searching against the library using RepeatMasker (v4.10)^26^. Ultimately, 366.98 Mb of TE sequences were identified, accounting for 52.75% of the genome assembly (Table 4). Long interspersed nuclear elements (LINE) were the largest category of transposable elements, representing 21.60% of the genome, followed by large retrotransposon derivatives (LARD), representing 8.84% of the genome (Table 4). Short interspersed nuclear elements (SINE), long terminal repeats (LTR), and DNA transposons accounting for 0.05%, 7.83%, and 11.46% of the whole genome, respectively (Table 4).

### Gene modeling and prediction

After removing the repeat sequences, we performed integrated prediction of intact protein-coding gene models using three independent approaches: *de novo* prediction, homology-based prediction, and transcript prediction. Augustus (v2.4)^30^ and SNAP^31^ were used for *de novo* prediction. Homology-based gene prediction was conducted using GeMoMa (v1.3.1)^32^ against the protein sequences of five insects, *Acyrthosiphon pisum*, *Bombyx mori*, *Bombus terrestris*, *Plutella xylostella*, and *Amyelois transitella*, downloaded from InsectBase 2.0^33^ (Supplementary Table 1). For transcriptome-based annotation, clean RNA-seq reads were aligned to the *S. incertulas* genome assembly using HISAT2 (v2.2.1)^34^ and the gene set was predicted using PASA (v2.3.2)^35^. Finally, the gene models obtained from these three methods were integrated into a unified gene set using EVidenceModeler (v1.1.1)^36^ with default parameters. Overall, 14,850 protein-coding genes were annotated in the *S. incertulas* genome (Table 5). To perform functional annotation of the protein-coding genes, we aligned the predicted genes against databases, including NR, KOG, KEGG, and TrEMBL, using BLAST (v2.2.31)^37^ with a threshold of 1e^−5^. In total, 14,148 genes, accounting for 95.27% of the predicted genes, were annotated in at least one database (Table 5). Furthermore, 7,446 genes were assigned to GO terms and 5,762 genes were mapped to at least one KEGG pathway (Table 5).

### Gene family identification

Given that host adaptation usually involves host recognition and detoxification of host secondary metabolites^38,39^, to investigate the potential reason for the specialized feeding preference of *S. incertulas* at the genomic level, we performed a comparative analysis of the detoxification- and chemosensory-related genes of *S. incertulas* and eight other Lepidoptera insects with different feeding habits. These species are classified as monophagous insects (*S. incertulas*, *Danaus plexippus*, and *B. mori*), oligophagous insects (*C. suppressalis, Manduca sexta*, and *P. xylostella*), and polyphagous insects (*Helicoverpa armigera, Spodoptera litura*, and *Spodoptera frugiperda*). The detoxification-related genes, P450, CCE, GST, ATP-binding cassette (ABC), and UDP-glycosyltransferases (UGT), as well as the chemosensory-related genes, GRs, ORs, OBPs, ionotropic receptors (IRs), and chemosensory proteins (CSPs) were further annotated using BLASTP (E < 10^−5^) (Fig. 5). Our results showed a positive relationship between the number of genes and the host range. We observed that polyphagous species had relatively high numbers of genes, especially in the detoxification-related (P450, CCE, and GST) and chemosensory-related (GR, OR, and OBP) gene families, which is consistent with the findings of previous studies^16^. Compared to *C. suppressalis*, *S. incertulas* had a lower number of GR, OR, and OBP genes, which may indicate that chemosensory processes play critical roles in determining the host preference of *S. incertulas*. The sample size for this analysis was quite small, and therefore, further studies are needed to determine the functions associated with these genes.

## Data Records

Raw Illumina, PacBio HiFi and Hi-C *S. incertulas* genome sequencing data were deposited in the NCBI SRA database with the accession number SRP47613^40^. The reference genome was deposited in the GenBank with the accession number JAYEAL000000000^41^. The annotation of the *S. incertulas* genome have been deposited at figshare24793869^42^.

## Technical Validation

### Evaluation of the genome assembly

Three independent methods were used to evaluate the accuracy and completeness of the *S. incertulas* genome assembly. First, clean reads acquired from Illumina sequencing were aligned against the genome assembly using BWA^23^. The results revealed that 98.28% of the Illumina reads aligned with the genome assembly. Second, the Core Eukaryotic Genes Mapping Approach database contained 458 conserved core eukaryotic genes, of which 403 (87.99%) were identified in the *S. incertulas* genome (Supplementary Table 2). Third, the completeness of the two rattan assemblies was evaluated using BUSCO (v2.5)^19^ from the insecta.odb10 database, which quantitatively assesses genome completeness based on evolutionarily informed expectations of gene content from near-universal single-copy orthologs. The BUSCO results showed that 97.65% (1,619/1,658) of the conserved BUSCO proteins were detected in the *S. incertulas* assembly, which was significantly higher than the 48.87% detected in the recent assembly version of *S. incertulas*^11^ (Table 2). Of the 1,658 single-copy orthologs, 95.60% were complete and single copy, 2.05% were complete and duplicated, 0.97% were fragmented, and 1.39% were missing (Table 6). To assess the quality of the chromosome assembly, the assembly was sheared into 100 kb bins, and the intensity of the interaction pairs was used to plot heatmaps. The Hi-C heatmap showed that the intensity of the interaction was higher along the diagonals than at the non-diagonal positions in 22 distinct chromosomes (Fig. 2b). These results indicated that we obtained high-quality *S. incertulas* genome assemblies.

**Table 6:** Statistical results of BUSCO evaluation analysis of the genome assembly.

	Gene Number	Percentage (%)
Complete BUSCOs (C)	1,619	97.65
Complete and single-copy BUSCOs (S)	1,585	95.60
Complete and duplicated BUSCOs (D)	34	2.05
Fragmented BUSCOs (F)	16	0.97
Missing BUSCOs (M)	23	1.39
Total BUSCO groups searched	1,658	100

### Genome synteny analysis

Genome synteny analysis of *S. incertulas* with another stem borer, *C. suppressalis*, was performed using TBtools-II (v2.019)^43^ (Fig. 3). The YSB genome showed high chromosomal synteny with *C. suppressalis*, and several fusion and fission events were also detected. The *S. incertulas* Chr1 was syntenic to the Chr2 and Chr22 of the *C. suppressalis*; the *S. incertulas* Chr2 was syntenic to a large portion of the Chr7 and a small fragment of Chr1 of the *C. suppressalis*.

### Comparative genomics and phylogenetic reconstruction

The protein sequences of 17 insects, including four insect orders (Lepidoptera, Hemiptera, Diptera, and Hymenoptera), were collected from InsectBase 2.0^33^ (Supplementary Table 1). Only the longest transcript of each gene was used for analysis. Single-copy orthologs among genomes of all species were determined using OrthoFinder (v2.4.0)^44^ with the default parameters. To infer the phylogeny of these insects, multiple sequence alignments of single-copy gene families were performed using MAFFT (v7.310)^45^ with the “-auto” parameter, and trimming was performed using Gblock (v0.91b)^46^ with the default setting. The alignment results were concatenated to construct a maximum likelihood phylogenetic tree using RAxML (v8)^47^ with 1,000 bootstrap replicates. The divergence time between different species was estimated using MCMCtree (PAML^48^ package) based on the fossil records acquired from the TimeTree database^49^ (http://www.timetree.org/) using the approximate likelihood calculation method (*A. mellifera* vs. *N. vitripennis* 162.4–219.3 Mya, *B. mori* vs. *M. sexta* 39.8–95.1 Mya, *D. plexippus* vs. *H. melpomene* 69.4–111.5 Mya, *H. armigera* vs. *S. frugiperda* 38.0–60.3 Mya, and *S. incertulas* vs. *A. gambiae* 151.9–344.7 Mya). A total of 22,069 orthologous gene families were identified from 18 insect species, of which 415 single-copy orthologous gene families were used for phylogenetic analysis (Fig. 4). The results of the phylogenetic analysis indicated that Lepidopteran insects speciated from their common ancestors later than Dipteran, Hymenopteran, and Hemipteran insects (Fig. 4). *S. incertulas* and *C. suppressalis* clustered into a single clade within Lepidoptera and diverged at approximately 72.64 Mya.

### Gene family expansion and contraction

Furthermore, the results obtained from phylogenetic trees, which included divergence time, were used to identify the expansion and contraction of gene families using CAFE (v5.0)^50^ with a *p*-value threshold <0.05 as the cut-off. Of the 22,069 gene families in the most recent common ancestor of all 18 species, 860 were expanded and 1,116 were contracted in *S. incertulas* (Fig. 4), and 49 expanded and 21 contracted families were identified (*p* < 0.05). GO enrichment analysis of the 49 expanded TreeFam families in *S. incertulas* showed that these genes were significantly enriched in defense responses to bacterium (GO:0042742, GO:0009617), biotic stimulus (GO:0009607, GO:0043207) and other organism (GO:0098542), which might play critical roles in increasing ecological adaptation and insecticide resistance. In addition, some genes were enriched in aromatic compound metabolic processes (GO:006725, GO:0019438), which may be associated with the metabolism of anthocyanins in the host plant (Supplementary Table 3). GO analysis demonstrated that 21 contracted TreeFam gene families were significantly enriched during cuticle development (GO:0008010, GO:0005214, GO:0042302, GO:0040003, and GO:0042335) (Supplementary Table 4). However, further investigations are needed to determine the functions associated with the genes in these expanded and contracted gene families, such as an analysis of their expression patterns and their putative roles in ecological-adaptation-associated processes.

### Supplementary information

Supplementary Information

## Data Availability

All software and pipelines were executed according to the manuals and protocols of published bioinformatics tools. The software version and code/parameters are described in the Methods section.
