# Supplementary material for: A chromosome-level genome assembly of yellow stem borer (Scirpophaga incertulas)
Source: Sci Data. 2024 Mar 8;11:279. doi: 10.1038/s41597-024-03108-3 (PMC10923946; doi:10.1038/s41597-024-03108-3)
Supplement: Supplementary file 1 — Supplementary Information [file 41597_2024_3108_MOESM1_ESM.docx]

**Supplementary Information**

**A chromosome-level genome assembly of yellow stem borer** **(*Scirpophaga incertulas*)**

**Sicong Zhou^1#^, Guanghua Luo^1#^, Qiong Yang^2^, Yangchun Han^1, 3^, Kaili Yuan^1^, Rui Ji^1, 4^* & Jichao Fang^1^***

^1^Institute of Plant Protection, Jiangsu Academy of Agricultural Sciences, Jiangsu Key Laboratory for Food and Safety-State Key Laboratory Cultivation Base of Ministry of Science and Technology, Nanjing 210014, China. ^2^Bio21 Institute, School of BioSciences, University of Melbourne, Parkville, Victoria 3010, Australia. ^3^Integrated Technical Service Center of Jiangyin Customs, Jiangyin 214441, China. ^4^Jiangsu Collaborative Innovation Center of Regional Modern Agriculture & Environmental Protection, Huaiyin Normal University, Huaian 223300, China. ^#^These authors contributed equally: Sicong Zhou, Guanghua Luo. *Corresponding authors: Rui Ji, jirui@jaas.ac.cn; Jichao Fang, fangjc@jaas.ac.cn.

**Contents**

| **Title** | **Page** |
| --- | --- |
| Supplementary Figure 1 | 2 |
| Supplementary Table 1 | 3 |
| Supplementary Table 2 | 4 |
| Supplementary Table 3 | 5 |
| Supplementary Table 4 | 8 |


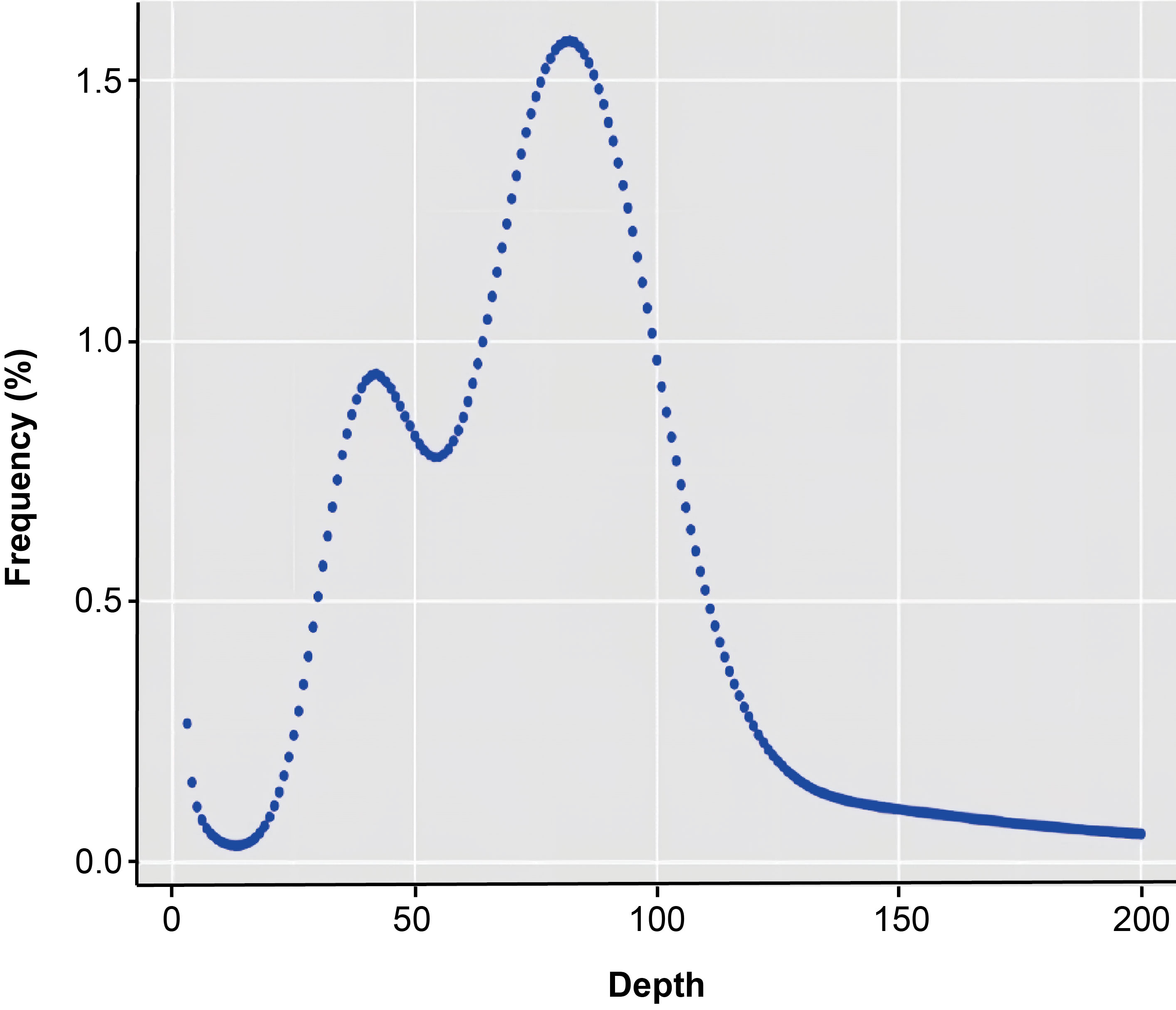


**Supplementary Figure 1 k-mer distribution (K = 19) of the *S. incertulas* genome.** The x-axis is the k-mer depth, and the y-axis represents the corresponding frequency of the k-mer at a given depth.

**Supplementary Table 1 Source of protein sequences of 21 insects.** Except for *S. incertulas* (this study), the protein sequences of the remaining 20 insects were downloaded from InsectBase 2.0 (<http://v2.insect-genome.com/>).

| **Species** | **Order** | **Accession** |
| --- | --- | --- |
| *Scripophage incertulas* | Lepidoptera | / |
| *Manduca sexta* | Lepidoptera | IBG_00533 |
| *Bombyx mori* | Lepidoptera | IBG_00145 |
| *Chilo suppressalis* | Lepidoptera | IBG_00177 |
| *Plutella xylostella* | Lepidoptera | IBG_00646 |
| *Heliconius melpomene* | Lepidoptera | IBG_00434 |
| *Danaus plexippus* | Lepidoptera | IBG_00230 |
| *Spodoptera litura* | Lepidoptera | IBG_00716 |
| *Spodoptera frugiperda* | Lepidoptera | IBG_00715 |
| *Helicoverpa armigera* | Lepidoptera | IBG_00442 |
| *Amyelois transitella* | Lepidoptera | IBG_00027 |
| *Nilaparvata lugens* | Hemiptera | IBG_00572 |
| *Laodelphax striatellus* | Hemiptera | IBG_00477 |
| *Acyrthosiphon pisum* | Hemiptera | IBG_00009 |
| *Bemisia tabaci* | Hemiptera | IBG_00110 |
| *Myzus persicae* | Hemiptera | IBG_00561 |
| *Apis mellifera* | Hymenoptera | IBG_00083 |
| *Nasonia vitripennis* | Hymenoptera | IBG_00564 |
| *Bombus terrestris* | Hymenoptera | IBG_00138 |
| *Drosophila melanogaster* | Diptera | IBG_00296 |
| *Anopheles gambiae* | Diptera | IBG_00050 |

**Supplementary Table 2** **Evaluation results of the genome set using the Core Eukaryotic Genes Mapping Approach database.**

|  | **Number^#^** | **Percentage (%)^*^** |
| --- | --- | --- |
| 458 core eukaryotic genes (CEGs) | 403 | 87.99 |
| 248 highly conserved CEGs | 179 | 72.18 |

^#^ The number of CEGs identified in the assembly

^*^ Percentage of CEGs present in the assembly

**Supplementary Table 3 Gene** **Ontology enrichment of** **significantly expanded genes in *S. incertulas*, (*p*-adjusted < 0.05, hypergeometric test, false discovery rate (FDR)-adjusted).** GeneRatio indicated the ratio of significantly expanded genes that are annotated in the GO term. BgRatio indicated the ratio of all genes that are annotated in the GO term.

| **GO ID** | **Description** | **GeneRatio** | **BgRatio** | **pvalue** | ***p*-adjusted** |
| --- | --- | --- | --- | --- | --- |
| GO:0140097 | catalytic activity, acting on DNA | 38/110 | 164/6266 | 0 | 0 |
| GO:0003676 | nucleic acid binding | 82/1175 | 164/6266 | 0 | 0 |
| GO:0004518 | nuclease activity | 22/80 | 164/6266 | 0 | 0 |
| GO:0004519 | endonuclease activity | 22/52 | 164/6266 | 0 | 0 |
| GO:0003964 | RNA-directed DNA polymerase activity | 38/47 | 164/6266 | 0 | 0 |
| GO:0034061 | DNA polymerase activity | 38/58 | 164/6266 | 1.11E-16 | 4.63E-16 |
| GO:0140640 | catalytic activity, acting on a nucleic acid | 38/311 | 164/6266 | 1.11E-16 | 4.63E-16 |
| GO:0003723 | RNA binding | 37/211 | 164/6266 | 2.22E-16 | 6.94E-16 |
| GO:0016779 | nucleotidyltransferase activity | 40/117 | 164/6266 | 2.22E-16 | 6.94E-16 |
| GO:0016772 | transferase activity, transferring phosphorus-containing groups | 40/540 | 164/6266 | 6.82E-10 | 1.70E-09 |
| GO:0003796 | lysozyme activity | 6/8 | 164/6266 | 7.87E-09 | 1.79E-08 |
| GO:0061783 | peptidoglycan muralytic activity | 6/11 | 164/6266 | 1.22E-07 | 2.53E-07 |
| GO:0016740 | transferase activity | 50/1044 | 164/6266 | 6.44E-06 | 1.24E-05 |
| GO:0016798 | hydrolase activity, acting on glycosyl bonds | 13/120 | 164/6266 | 1.30E-05 | 2.32E-05 |
| GO:0005488 | binding | 119/3554 | 164/6266 | 1.60E-05 | 2.66E-05 |
| GO:0097159 | organic cyclic compound binding | 82/2140 | 164/6266 | 1.65E-05 | 2.58E-05 |
| GO:0016788 | hydrolase activity, acting on ester bonds | 22/316 | 164/6266 | 2.16E-05 | 3.18E-05 |
| GO:0004553 | hydrolase activity, hydrolyzing O-glycosyl compounds | 6/93 | 164/6266 | 0.0344898 | 0.0479026 |
| GO:0006278 | RNA-templated DNA biosynthetic process | 37/45 | 100/5239 | 0 | 0 |
| GO:0046483 | heterocycle metabolic process | 76/1118 | 100/5239 | 0 | 0 |
| GO:0043170 | macromolecule metabolic process | 86/1978 | 100/5239 | 0 | 0 |
| GO:0090304 | nucleic acid metabolic process | 76/916 | 100/5239 | 0 | 0 |
| GO:0034641 | cellular nitrogen compound metabolic process | 76/1347 | 100/5239 | 0 | 0 |
| GO:0008152 | metabolic process | 97/073 | 100/5239 | 0 | 0 |
| GO:0071897 | DNA biosynthetic process | 37/52 | 100/5239 | 0 | 0 |
| GO:0006259 | DNA metabolic process | 76/249 | 100/5239 | 0 | 0 |
| GO:0006139 | nucleobase-containing compound metabolic process | 76/1078 | 100/5239 | 0 | 0 |
| GO:1901360 | organic cyclic compound metabolic process | 76/1155 | 100/5239 | 1.11E-16 | 8.55E-16 |
| GO:0006725 | cellular aromatic compound metabolic process | 76/1132 | 100/5239 | 2.22E-16 | 1.55E-15 |
| GO:0071704 | organic substance metabolic process | 86/2615 | 100/5239 | 2.18E-14 | 1.40E-13 |
| GO:0044237 | cellular metabolic process | 76/2181 | 100/5239 | 1.86E-12 | 1.10E-11 |
| GO:0044238 | primary metabolic process | 81/477 | 100/5239 | 2.49E-12 | 1.37E-11 |
| GO:0006807 | nitrogen compound metabolic process | 76/2224 | 100/5239 | 6.03E-12 | 3.10E-11 |
| GO:0034654 | nucleobase-containing compound biosynthetic process | 37/618 | 100/5239 | 4.05E-11 | 1.95E-10 |
| GO:0019438 | aromatic compound biosynthetic process | 37/651 | 100/5239 | 1.89E-10 | 8.58E-10 |
| GO:0018130 | heterocycle biosynthetic process | 37/657 | 100/5239 | 2.48E-10 | 1.06E-09 |
| GO:1901362 | organic cyclic compound biosynthetic process | 37/671 | 100/5239 | 4.59E-10 | 1.86E-09 |
| GO:0009987 | cellular process | 94/3745 | 100/5239 | 1.18E-08 | 4.52E-08 |
| GO:0019835 | cytolysis | 5/6 | 100/5239 | 1.36E-08 | 4.97E-08 |
| GO:0071554 | cell wall organization or biogenesis | 5/8 | 100/5239 | 1.23E-07 | 4.29E-07 |
| GO:0044036 | cell wall macromolecule metabolic process | 5/8 | 100/5239 | 1.23E-07 | 4.29E-07 |
| GO:0016998 | cell wall macromolecule catabolic process | 5/8 | 100/5239 | 1.23E-07 | 4.29E-07 |
| GO:0044271 | cellular nitrogen compound biosynthetic process | 37/933 | 100/5239 | 3.35E-06 | 1.03E-05 |
| GO:0009059 | macromolecule biosynthetic process | 37/1072 | 100/5239 | 8.66E-05 | 2.47E-04 |
| GO:0042742 | defense response to bacterium | 5/36 | 100/5239 | 5.40E-04 | 0.0014855 |
| GO:0009617 | response to bacterium | 5/38 | 100/5239 | 6.98E-04 | 0.0018528 |
| GO:0098542 | defense response to other organism | 6/75 | 100/5239 | 0.0028931 | 0.0074255 |
| GO:0009607 | response to biotic stimulus | 6/77 | 100/5239 | 0.0033038 | 0.0079499 |
| GO:0043207 | response to external biotic stimulus | 6/77 | 100/5239 | 0.0033038 | 0.0079499 |
| GO:0051707 | response to other organism | 6/77 | 100/5239 | 0.0033038 | 0.0079499 |
| GO:0044419 | biological process involved in interspecies interaction between organisms | 6/79 | 100/5239 | 0.0037567 | 0.0082647 |
| GO:0006952 | defense response | 6/81 | 100/5239 | 0.0042542 | 0.0088533 |
| GO:0044249 | cellular biosynthetic process | 37/1306 | 100/5239 | 0.0045631 | 0.0090092 |
| GO:1901576 | organic substance biosynthetic process | 37/1330 | 100/5239 | 0.0062855 | 0.0118046 |
| GO:0009058 | biosynthetic process | 37/1351 | 100/5239 | 0.0082236 | 0.0150765 |
| GO:0008150 | biological process | 100/5032 | 100/5239 | 0.0170656 | 0.0298648 |

**Supplementary Table 4 Gene Ontology enrichment of significantly** **contracted genes in *S. incertulas* (*p*-adjusted < 0.05, hypergeometric test, FDR-adjusted).** GeneRatio indicated the ratio of significantly contracted genes that are annotated in the GO term. BgRatio indicated the ratio of all genes that are annotated in the GO term.

| **GO ID** | **Description** | **GeneRatio** | **BgRatio** | **pvalue** | ***p*-adjusted** |
| --- | --- | --- | --- | --- | --- |
| GO:0008010 | structural constituent of chitin-based larval cuticle | 14/42 | 14/6777 | 0 | 0 |
| GO:0005198 | structural molecule activity | 14/421 | 14/6777 | 0 | 0 |
| GO:0005214 | structural constituent of chitin-based cuticle | 14/47 | 14/6777 | 2.22E-16 | 3.70E-16 |
| GO:0042302 | structural constituent of cuticle | 14/47 | 14/6777 | 2.22E-16 | 3.70E-16 |
| GO:0031012 | extracellular matrix | 14/146 | 14/7444 | 0 | 0 |
| GO:0030312 | external encapsulating structure | 14/156 | 14/7444 | 0 | 0 |
| GO:0044421 | obsolete extracellular region part | 14/617 | 14/7444 | 2.22E-16 | 5.18E-16 |
| GO:0005576 | extracellular region | 14/904 | 14/7444 | 1.38E-13 | 2.42E-13 |
| GO:0071944 | cell periphery | 14/2127 | 14/7444 | 2.35E-08 | 3.28E-08 |
| GO:0040003 | chitin-based cuticle development | 14/111 | 14/7717 | 0 | 0 |
| GO:0042335 | cuticle development | 14/192 | 14/7717 | 0 | 0 |
| GO:0007275 | multicellular organism development | 14/3663 | 14/7717 | 2.91E-05 | 6.79E-05 |
| GO:0048856 | anatomical structure development | 14/3916 | 14/7717 | 7.42E-05 | 1.30E-04 |
| GO:0032502 | developmental process | 14/4109 | 14/7717 | 1.46E-04 | 2.04E-04 |
| GO:0032501 | multicellular organismal process | 14/4655 | 14/7717 | 8.38E-04 | 9.78E-04 |
